# Enabling WLAN and WPAN Coexistence via Cross-Technology Communication

**DOI:** 10.3390/s22030707

**Published:** 2022-01-18

**Authors:** Seungku Kim

**Affiliations:** Department of Electronics Engineering, Chungbuk National University, Cheongju-si 28644, Korea; kimsk@cbnu.ac.kr; Tel.: +82-43-261-2479

**Keywords:** coexistence, cross-technology communication, WLAN, WPAN, 2.4 GHz

## Abstract

In the limited frequency spectrum shared by various wireless communication technologies, cross-technology interference is an important factor which determines communication performance. A variety of coexistence methods to reduce the impact of this interference have been studied, but most of them cannot explicitly coordinate the shared spectrum and are not practical. This paper presents an explicit coexistence mechanism using cross-technology communication among heterogeneous wireless technologies. This mimics “carrier sense multiple access with collision avoidance” (CSMA/CA) via bidirectional cross-technology communication, which is called CTC-CSMA/CA. It allows communication between heterogeneous wireless technologies in order to achieve CSMA/CA. This accurately assigns required channel resources by directly sending and receiving feedback. CTC-CSMA/CA is a highly compatible technology because it does not require any modification to the IEEE 802.11 standard or any extra hardware. In addition, Zigbee can operate with a low duty cycle by synchronizing it to a periodic Wi-Fi beacon. We implemented CTC-CSMA/CA using a commodity Wi-Fi access point and a commercial Zigbee platform. Our experiments showed that the channels are coordinated more accurately by our method, which significantly improves Zigbee throughput, than by conventional schemes. We expect the proposed scheme to be an important application case in designing future cross-technology communication.

## 1. Introduction

The Internet of Things (IoT) is a system that provides services by connecting various electronic devices to the Internet. With the increase in IoT devices, the number of devices using wireless communication is increasing exponentially. These IoT devices share a limited frequency spectrum, which causes interference. In the 2.4-GHz industrial, scientific, and medical (ISM) band, various communication technologies, such as Wi-Fi, Bluetooth, Zigbee, Thread, etc., share the frequency band. Each communication technology provides protocols for sharing the same channel among wireless devices, but this is limited to other devices which have the same technology. Performance degradation still occurs due to cross-technology interference (CTI). In an overcrowded spectrum (e.g., ISM), coexistence among cross-technology devices should be considered.

Conventional studies and standards present channel coordination mechanisms. Most works [1] use implicit channel coordination schemes based on statistical models, which do not guarantee complete interference avoidance. Several studies [2,3] proposed explicit channel coordination using extra hardware, but this is not practical. Z. Yin et al. [4] proposed an explicit channel coordination method (ECC) using cross-technology communication (CTC) without extra hardware. CTC enables direct communication between wireless devices using incompatible physical layers. ECC is the only technique that coordinates the shared spectrum explicitly via CTC. ECC significantly reduces the impact of CTI by securing an interference-free period, that is, white space. Wi-Fi determines the white space and delivers it to Zigbee via CTC. However, the white space is determined statistically by Wi-Fi without any feedback from Zigbee. This scheme still needs improvement.

The accuracy of the white space estimation based on the existing statistical model differs depending on the communication environment. We coordinate a shared channel more explicitly by exchanging feedback using CTC. This paper also presents an explicit coexistence mechanism between wireless local area networks (WLANs) and wireless personal area networks (WPANs) via CTC, such as ECC. In this paper, Wi-Fi and Zigbee are used as the WLAN and WPAN technologies, respectively. This can be extended to other technologies. The proposed mechanism mimics “carrier sense multiple access with collision avoidance” (CSMA/CA), a typical channel-sharing scheme, via CTC, that is, CTC-CSMA/CA. The novelty of CTC-CSMA/CA is that Zigbee and Wi-Fi agree to use the shared channel via CTC-based bidirectional communication. To the best of our knowledge, this has not been tried yet. This enables Wi-Fi to receive a request-to-send (RTS) frame from Zigbee, and Wi-Fi to send a clear-to-send (CTS) frame to Zigbee. Wi-Fi AP allocates an accurate white space by receiving direct feedback (e.g., an RTS) from Zigbee in traffic dynamics, rather than using a statistical model, as in ECC. This makes Zigbee transparent to Wi-Fi. All Zigbee devices using CTC-CSMA/CA are synchronized with periodic beacons from a Wi-Fi AP. This allows Zigbee to ensure a low duty cycle. We implemented CTC-CSMA/CA and compared it with the legacy channel coordination models. As a result of the experiment, when Zigbee transmits data at 50 Kbps, CTC-CSMA/CA secures an additional white space of 77.47% compared to ECC. This is due to the difference between the statistical model of ECC and the direct coordination of CTC-CSMA/CA when determining the white space. ECC shows lower throughput compared to channel coordination methods in homogeneous wireless technology. This result shows an error of statistical model-based white space inference. CTC-CSMA/CA shows a throughput gain of 70.62% compared to ECC, and shows an average throughput gain of 20% or more compared to the traditional channel coordination mechanisms. This shows that accurate white space inference is important. Our primary contributions are summarized as follows:

- We enable CSMA/CA between heterogeneous wireless technologies by using cross-technology communication between WLANs and WPANs. This allows more accurate channel coordination than statistical and probabilistic model-based channel coordination.
- The proposed method does not require hardware and standard modification for either WLANs or WPANs. Hence, it is a practical and scalable technology applicable to other wireless communication systems.
- We implement a real testbed on commodity Wi-Fi APs and Zigbee devices. In the experimental result, it shows packet reception ratio of 97.48% and improves by throughput of 70.62% compared to the recent channel coordination system, ECC.

The rest of this paper is organized as follows. Section 2 introduces the background and related works. In Section 3, we present the proposed coexistence mechanism. Section 4 evaluates the performance of the proposed protocol, and conclusions are drawn in Section 5.

## 2. Background and Related Works

IoT services have resulted in an increase in wireless communication devices. This has caused crowding in limited frequency bands, especially in the industry, scientific, and medical (ISM) band. The 2.4-GHz ISM band, which is widely used, is shared by various devices, such as Wi-Fi, Bluetooth, Zigbee, microwaves, and cordless phones. This section introduces several related studies on sharing the limited spectrum in the 2.4-GHz ISM band.

### 2.1. Channel Coordination in WLANs and WPANs

The IEEE 802.11 MAC (media access control) [5] sublayer provides two fundamental channel-access mechanisms: a mandatory distributed coordination function (DCF), and an optional point coordination function (PCF) [6]. DCF is contention-based MAC protocol that uses CSMA/CA. CSMA/CA uses a “listen before talk” channel-access strategy. The sender performs clear channel assessment (CCA) and exchanges RTS, CTS, and data frames if the channel is idle during DCF inter-frame space. The IEEE 802.11 stations that overhear RTS and CTS frames are set to “network allocation vector” (NAV) for the time specified in the duration field. The NAV is a virtual carrier-sensing mechanism intended to maintain an idle channel for a fixed duration. If the channel is not idle in the CCA, or RTS, CTS, and data transmission fail, the sender tries CCA again after a random backoff. PCF is a contention-free-based MAC protocol that runs on an infrastructure network. A point coordinator (i.e., access point) grants transmission rights to each station using a polling mechanism. If the optional PCF is used, the superframe is shared with the mandatory DCF. Most off-the-shelf APs operate only with DCF.

WPANs are a low-cost, low-power, and short-range wireless communication technology that is expected to be used in many IoT devices. Bluetooth [7] and IEEE 802.15.4 [8] are the most widely used WPAN technologies. The Bluetooth 1.0 standard was released in 1999, and the Bluetooth 5.0 standard is now commercially available. It uses an adaptive frequency hopping scheme that randomly hops the channels 1600 times per second to reduce co-channel interference. IEEE 802.15.4 MAC supports unslotted CSMA/CA for channel coordination. Time-division-multiple-access-based slotted CSMA/CA can be optionally used, according to application requirements.

### 2.2. Coexistence for Heterogeneous Wireless Networks

In the 2.4-GHz band, various types of wireless communication devices share a limited spectrum, and their numbers are expected to increase in the future. Coexistence of wireless communication technologies in the limited spectrum is important for improving network performance. Wireless communication standards are designed to be interoperable with homogeneous wireless communication technologies, but do not consider coexistence with heterogeneous wireless technologies. Since heterogeneous wireless technologies use different physical layers, it is difficult to coordinate the shared channels with each other.

IEEE 802.11 and IEEE 802.15.4 allow access to channels using CSMA/CA. Carrier sensing determines the channel state via signal strength, thus the CSMA/CA can coordinate channel access among heterogeneous wireless networks through the backoff algorithm. However, IEEE 802.11 cannot detect the IEEE 802.15.4 signal due to the differences in bandwidth and transmission power between the two technologies. This causes IEEE 802.11 to interfere with IEEE 802.15.4. Bluetooth is not significantly affected by interference, because it uses adaptive frequency hopping, therefore we do not consider the coexistence of Bluetooth in this study.

IEEE 802.15.2-2003 [9] recommends practices for the coexistence of WLAN and WPAN. This standard developed a coexistence model and a set of coexistence mechanism between Bluetooth and WLAN. IEEE 802.19 [10] has extended the developing scope for coexistence to the IEEE 802 standards of unlicensed bands, such as IEEE 802.11 WLAN, IEEE 802.15 WPAN, IEEE 802.16 wireless metropolitan area networks (WMANs), and IEEE 802.22 wireless regional area networks (WRANs).

D. Yang et al. [1] categorized the conventional coexistence works into three types of solution: frequency sharing, time sharing, and space sharing. In frequency-sharing solutions, the victim or interferer changes the channel if interference is detected. The time-sharing solution controls time intervals and traffic by cooperation between the victim and interferer. In a space-sharing solution, transmission power and CCA threshold control are used to avoid interference. The introduced works use implicit channel-coordination mechanisms that provide probabilistic models to avoid CTI. However, implicit channel coordination has inherent uncertainties due to channel dynamics [4].

CBT [2] and CCS [3] proposed explicit interference avoidance mechanisms using cooperative signaling. Zigbee uses a dedicated signaler that intentionally emits a busy tone when the Zigbee network needs to access the channel. The Zigbee signaler uses a higher output power than normal Zigbee transmitters in order to enhance visibility to Wi-Fi, and temporally hops to an adjacent channel in order not to affect interference to the Zigbee network. This scheme significantly reduces the effects of interference, but it requires extra hardware just for the channel coordination, and causes spectrum waste by signaling and Wi-Fi random backoff. Moreover, the 20-dBm output power used by the Zigbee signaler is not supported by IEEE 802.15.4 wireless communication devices. AA CTS-blocking [11] is a method in which the Wi-Fi AP secures a Zigbee channel through CTS frame transmission. A Zigbee coordinator must be wired to a Wi-Fi station and can transmit RTS frames via this Wi-Fi station. This provides explicit coexistence between Zigbee and Wi-Fi, but it is not practical in terms of wiring the Zigbee coordinator and Wi-Fi station.

### 2.3. Cross-Technology Communication

Recently, extensive research has been actively carried out concerning CTC that enables the exchange of information between heterogeneous wireless communication devices using incompatible physical layers. In this section, we introduce CTC and several related works. The proposed CTC can be categorized into two types: packet-level CTCs, and physical-level CTCs. Wireless communication devices can sense the energy of a signal emitted by an incompatible physical layer if the frequency bands overlap.

The packet-level CTC delivers symbols to heterogeneous wireless communication devices using this characteristic. Esense [12] embeds symbols in a signal length. The IEEE 802.11 WLAN transmitter varies the packet size to produce appropriate energy burst durations, which the receiver interprets as symbol. In ToneSense [13] and WiZig [14], the transmitter uses different output power levels to encode the symbols, and the receiver interprets the signal strength to decode the symbols. Gap Sense [15] embeds symbols in signal timing. Gap Sense adjusts the pulse timing of the preamble, and FreeBee [16] controls the beacon interval according to the symbols. C-Morse [17] and DCTC [18] encode symbols using different signal patterns. The transmitter generates a signal pattern by adjusting the release time of existing packets in the buffer. ZigFi [19] first introduces the CTC from Zigbee to Wi-Fi. Although FreeBee and DCTC provide the results of Zigbee to Wi-Fi communication, a detailed mechanism is not explained in the literature. As described in [19], Zigbee signals are weak and susceptible to noise at a Wi-Fi receiver because the bandwidth is much narrower and the output power is lower for Zigbee than for Wi-Fi. ZigFi uses channel state information, and piggybacks Zigbee packets over Wi-Fi packets for Zigbee-to-Wi-Fi CTC. Bsense [20] encodes symbols into a Wi-Fi beacon. Since the beacon periodically transmitted by the Wi-Fi AP is used for CTC, Bsense requires little overhead. In addition, Zigbee synchronizes to periodic beacon frames to maintain a very low duty cycle.

The physical-level CTC enables sending waveforms directly across incompatible physical layers via physical layer emulation. WEBee [21] and TwinBee [22] emulate Wi-Fi frames as Zigbee signals, and achieve much higher throughput than packet-level CTC. They can transfer information from Wi-Fi to Zigbee, but not vice versa. Therefore, physical-level CTC is not suitable for applications where Zigbee delivers information to Wi-Fi. ECC [4] proposed explicit channel coordination through physical-level CTC. ECC sends a Wi-Fi CTS frame on behalf of Zigbee, and then notifies Zigbee of the duration of the white space via physical-level CTC. During the white space, Zigbee can communicate without being affected by Wi-Fi interference. ECC allocates the white space from Wi-Fi to Zigbee considering only the Wi-Fi traffic status. ECC thus cannot determine when Zigbee needs to access the channel or what white-space duration is required. ECC supports a low duty cycle at the Zigbee. For this, Zigbee must maintain synchronization with ECC, but the literature does not consider the method or the cost of synchronization.

## 3. CTC-CSMA/CA

In this paper, we propose CTC-based CSMA/CA (CTC-CSMA/CA), which enables explicit channel coordination among heterogeneous wireless technologies. The design objective is to enable bidirectional communication across cross-technology devices without extra hardware and with low duty cycled Zigbee. This is designed to be compatible with the legacy CSMA/CA algorithm in IEEE 802.11.

### 3.1. Overview

**Figure 1:**
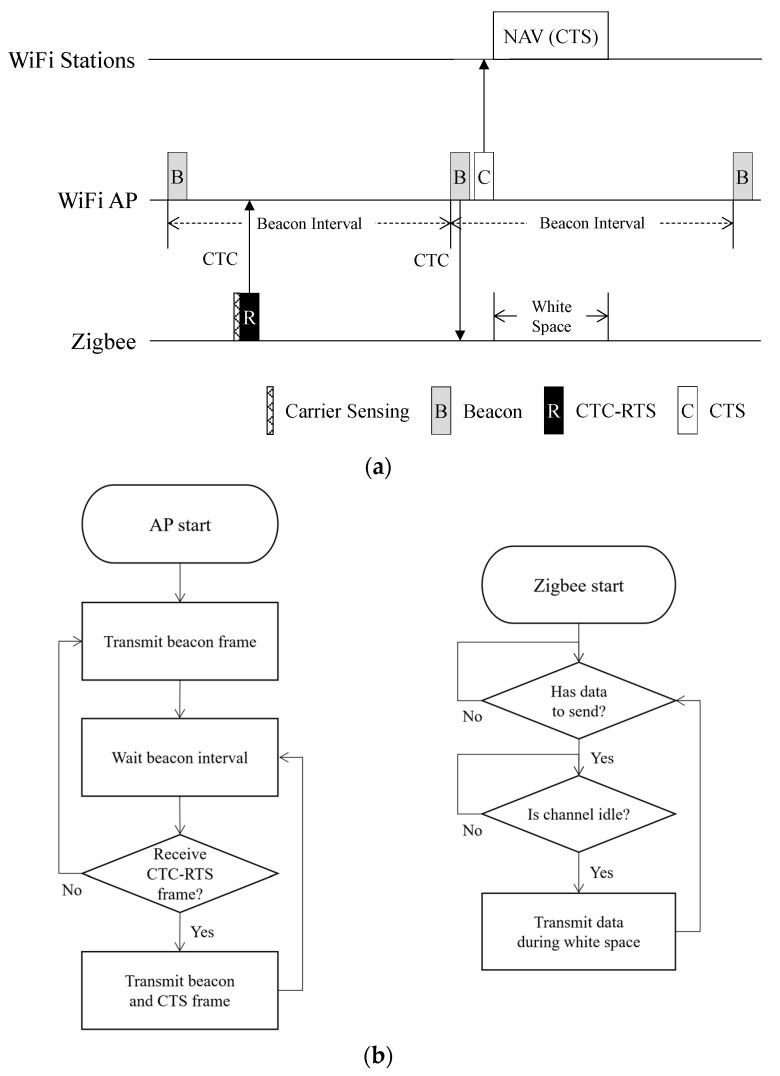
Overview of CTC−CSMA/CA Process. (**a**) Basic operation of CTC−CSMA/CA, (**b**) Flowchart of CTC−CSMA/CA.

Figure 1 shows the overview of channel coordination between Wi-Fi and Zigbee through CTC-CSMA/CA. A Zigbee device sends a request to a Wi-Fi AP for accessing the channel. Simce the Wi-Fi AP and the Zigbee device use incompatible physical layers, the Zigbee transmits a CTC-based RTS (CTC-RTS) frame. In IEEE 802.11 CSMA/CA, a Wi-Fi station uses a two-byte duration field in the RTS frame to request the required access time to the Wi-Fi AP. In CTC-CSMA/CA, Zigbee transmits a CTC-RTS frame including the required access time, that is, white space length. The white space refers to a section in which communication is possible without interference in a channel. Before transmitting the CTC-RTS frame, the Zigbee performs carrier sensing and waits until the channel becomes idle to avoid frame loss. The Wi-Fi AP receiving the CTC-RTS frame determines channel allocation by considering the Wi-Fi channel state. The Wi-Fi AP responds as to whether the channel is allowed via the next beacon frame. Bsense [20] is used to transmit this simple information to the Zigbee via a Wi-Fi beacon frame. After transmitting the beacon frame, the Wi-Fi AP sends a CTS frame to the Zigbee to secure white space. This CTS frame is delivered to nearby Wi-Fi stations, and they remain idle using the NAV setting. Zigbee is free from Wi-Fi interference during this white space.

### 3.2. Design of the CTC-RTS Frame

In IEEE 802.11 CSMA/CA, the RTS frame includes a two-byte duration field. The maximum allowable duration with the RTS duration field is 32 ms, and can be defined in units of 1 μs per bit. However, in the case of Zigbee, it is unnecessary to request white space in 1 μs increments due to its low data rate. The channel occupancy time of 32-byte Zigbee packet is 1 ms. Therefore, CTC-CSMA/CA requests white space in 1 ms increments, which requires six bits of the CTC-RTS frame.

It is important to deliver a 6-bit CTC-RTS frame reliably rather than quickly. Physical-level CTC achieves very high throughput compared to packet-level CTC, but it is a complex and error-prone mechanism. In addition, the physical-level CTC currently only provides the transmission from Wi-Fi to Zigbee. Hence, we designed the CTC-RTS frame based on packet-level CTC, which is much simpler and less error-prone than physical-level CTC. Packet-level CTC can embed symbols in signal length, strength, and timing. Since the signal strength is inherently changeable depending on characteristics of the communication environment, such as distance, obstacles, output power, etc. [20], the proposed CTC-RTS frame uses the signal length and timing.

**Figure 2:**
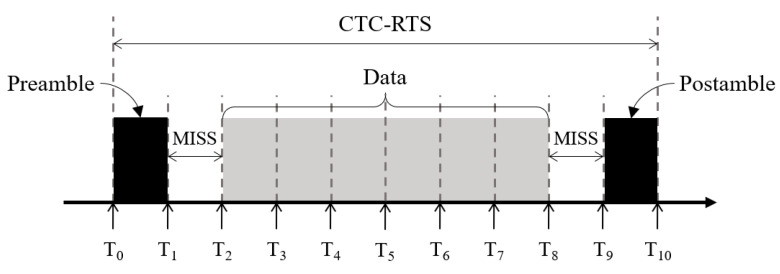
Constitution of the CTC-RTS frame.

As shown in Figure 2, the CTC-RTS frame is divided into 10 slots and consists of three parts: preamble, data, and postamble. The minimum length of a slot, Tn–Tn-1, depends on the sampling rate at the Wi-Fi AP. The preamble slot is always active to notify the Wi-Fi AP of the start of the CTC-RTS frame. Minimum inter-slot space (MISS) refers to the minimum idle time for the Wi-Fi AP to distinguish between the preamble and the data. After the MISS, the data signal is transferred from T2 to T8. The data slots contain the information about the six-bit required access time for the Zigbee network. They are composed of six slots with an active signal or an inactive signal according to the bit information. The active signal and the inactive signal represent 1 and 0, respectively. After the second MISS, the Zigbee activates the postamble slot, indicating the end of the CTC-RTS frame.

Wi-Fi communicates via wideband (20 MHz) and with a high transmission power (typically 100 mW), and Zigbee performs communication in narrowband (2 MHz) with a low transmission power (typically 1 mW). This asymmetry makes it difficult for Wi-Fi to discriminate the Zigbee signal from noise via RSSI [19]. OFDM-based systems divide a given channel into several narrow orthogonal subcarriers. In the 2.4 GHz band, IEEE 802.11n divides the 20 MHz and 40 MHz channels into 56 and 128 subcarriers, respectively. Each of these subcarriers has a 312.5 KHz band. We measure the magnitude of the subcarriers overlapping the Zigbee channel to identify the signal pattern of the CTS-RTS frame at the Wi-Fi AP. The spectral scan [23] is a custom feature in Atheros and Qualcomm chipsets, which enables scanning any wireless signals, including non-IEEE 802.11 preambles, in multiple frequency ranges. It provides fast Fourier transform (FFT) data from the baseband. We use the spectral scan to obtain an absolute magnitude of all subcarriers from the FFT data.

The sampling rate of the spectral scan can be defined as follows:

*R* = *P* × 256 × *f_s_* (1)

where *f_s_* is 44 MHz and 88 MHz for the 20 MHz and 40 MHz channels, respectively. *P* is a settable period ranging from 1 to 255. We can adjust the period between successive spectral scans by selecting *P*. The sampling time should be at least 4 μs; this is a minimum time for the PHY to pass FFT frames to MAC. Thus, the smallest Zigbee frame to activate a slot is one bit, based on a 250 Kbps data rate.

**Figure 3:**
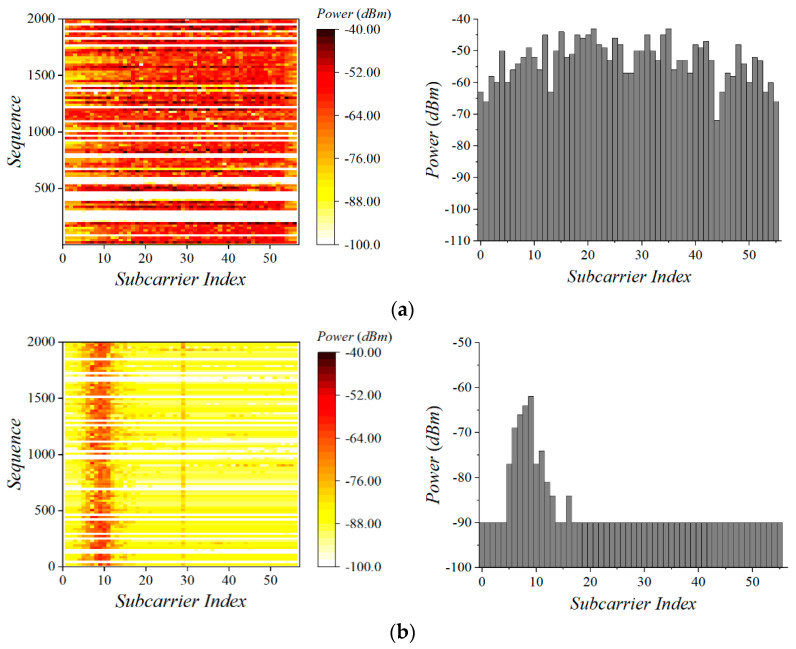
Monitored absolute magnitude of Wi−Fi and Zigbee traffic at the Wi−Fi AP. (**a**) Wi−Fi traffic, (**b**) Zigbee traffic.

Figure 3 presents the absolute magnitude of all Wi-Fi subcarriers when Wi-Fi stations and Zigbee devices transmit burst traffic. Wi-Fi distributes power evenly across all subcarriers. On the contrary, Zigbee shows magnitudes above the threshold only on the specific subcarriers. In this experiment, we used Wi-Fi channel 1 and Zigbee channel 13. Theoretically, the number of overlapping subcarriers is seven, but power above the noise floor is detected in 6–15 subcarriers due to an error. We use these characteristics to distinguish the CTC-RTS frame from other frames and noise at the Wi-Fi AP. The Wi-Fi AP monitors the channel using the background mode of the spectral scan. The background mode is running while the hardware is not busy with sending and receiving [23]. If the Wi-Fi AP recognizes the Zigbee signal pattern during continuous monitoring, it decodes the CTC-RTS frame. This is expected to enhance the reliability of the RSSI-based CTC from Zigbee to Wi-Fi.

### 3.3. White Space Allocation Mechanism

The Wi-Fi AP receiving the CTC-RTS frame determines whether the requested white space is allocated by considering the traffic state of the current Wi-Fi network. If the Wi-Fi AP allows the requested white space, it responds with the length of the white space allowed through the beacon frame. For this, we use Bsense [20], a CTC mechanism, which embeds the symbol into the Wi-Fi beacon frame for the CTC. It takes advantage of the fact that Zigbee can distinguish the energy length and intensity of the Wi-Fi signal. It is a highly practical CTC technology and offers a low data rate but little overhead. Bsense is a highly suitable technology for CTC-CSMA/CA since we aim to transmit simple six-bit white space lengths. All Zigbee devices are synchronized to the periodically transmitted Wi-Fi beacon frame. They periodically wake up and check whether white space is allowed or not.

If the white space is allowed, the Wi-Fi AP broadcasts a CTS frame containing the white space duration after the beacon frame. This prohibits Wi-Fi transmission and enables Zigbee to be free of interference during the white space. Typically, the CTS frame is 14 bytes is transmitted at 1 Mbps. The length of the active signal is fixed to 336 μs, including PLCP preamble and header. Zigbee can secure white space after the idle time of the short interframe space (10 μs in IEEE 802.11n 2.4 GHz) and the active time of the CTS frame (336 μs).

If the channel is insufficient, due to high Wi-Fi traffic, the Wi-Fi AP may deny the white space assignment to Zigbee. The rejection is also transferred through the Wi-Fi beacon frame with zero white space, and the CTS frame is not transmitted. In this case, the Zigbee retransmits the CTC-RTS frame including unallocated white space until Wi-Fi can afford.

## 4. Performance Evaluation

In this section, we evaluate the performance of the traffic load of Wi-Fi and Zigbee. In total, four modes were compared for our performance evaluation: continuous, CSMA/CA, ECC, and CTC-CSMA/CA. In the experiment, Wi-Fi was always operated based on IEEE 802.11 CSMA/CA. In the case of the continuous mode, Zigbee transmitted packets continuously without any coexistence method. In the CSMA/CA mode, Zigbee transmitted packets based on IEEE 802.15.4 CSMA/CA. The ECC mode is the only explicit channel coordination mechanism based on CTC. The CTC-CSMA/CA mode is our proposed protocol.

**Figure 4:**
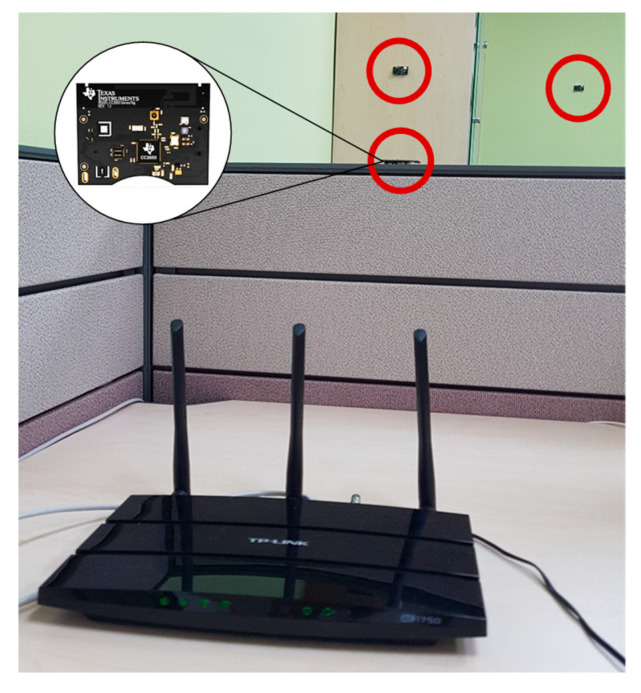
Experimental setup for performance evaluation.

Figure 4 shows our experimental setup. The Wi-Fi AP is the dual-band router TP-LINK AR1750 v2, which supports IEEE 802.11a/b/g/n/ac. IEEE 802.11n was used for generating traffic load. We ported an OpenWRT platform [24], which is a Linux-based open-source operating system, on the Wi-Fi AP. For the continuous and CSMA/CA tests, Wi-Fi AP operated as shipped. In the ECC experiment, the traffic rate was calculated for each beacon interval to determine the white space in the next beacon interval. The white space can be allocated for each beacon interval. We set the beacon interval to 100 TU (i.e., 102.4 ms) which is a commonly used value in most commodity Wi-Fi APs. For the CTC-CSMA/CA experiment, ath9k spectral scan [23] was used to receive the CTC-RTS frame from Zigbee. The sampling rate of the spectral scan, R, was 360,448 MHz, and PHY passed FFT frames to MAC every 8 μs. Bsense-L [20] was implemented on the Wi-Fi AP, to inform Zigbee of the length of the white space allowed, via a beacon frame. When the white space is allowed, the Wi-Fi AP transfers a CTS frame via the IEEE 802.11 CTS-to-Self protocol.

For the Zigbee nodes, we used the TI CC2650 SensorTag [25], which is a multi-standard platform supporting Bluetooth low energy and IEEE 802.15.4. We set the Zigbee channel to overlap with the Wi-Fi channel. Tx power was set to 5 dBm. In continuous mode, packets are transmitted periodically according to the transmission speed. The CSMA/CA mode also periodically transmits packets but performs IEEE 802.15.4 CSMA/CA operations before packet transmission. In the ECC and CTC-CSMA/CA modes, Zigbee nodes only transmit packets during the approved white space. The maximum allowable white space is assumed to be 32 ms, which is the maximum duration allowed in a Wi-Fi CTS frame.

### 4.1. Reliability of Zigbee Transmission

**Figure 5:**
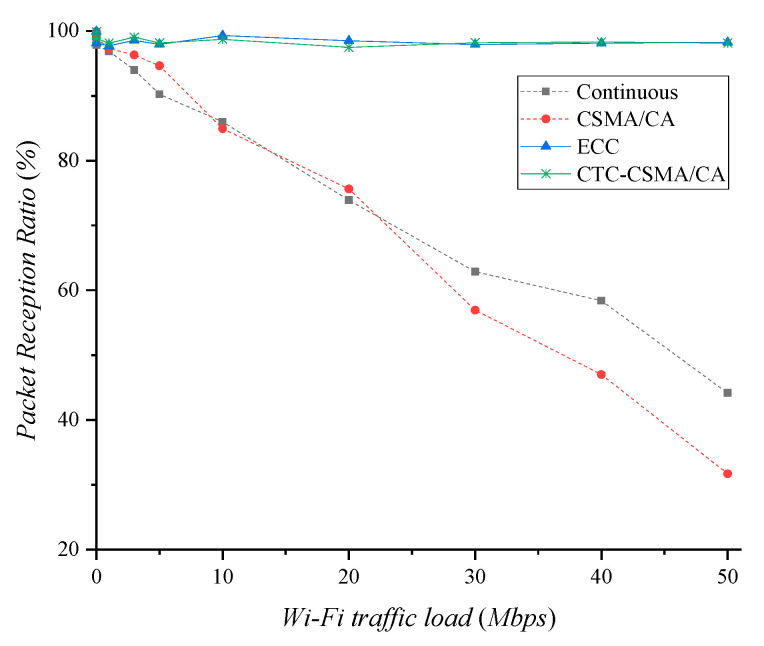
Packet reception ratio of Zigbee transmission according to Wi−Fi traffic load.

In this section, we discuss the experimental results to investigate effect of Wi-Fi signals on the Zigbee transmission. Figure 5 presents the packet reception ratio (PRR) of Zigbee. In this experiment, Zigbee transmitted at 50 Kbps, and Wi-Fi generated traffic of various capacities. We used iperf [26] to generate various Wi-Fi traffic loads. The aperiodic level of Wi-Fi traffic is determined by the standard deviation in latency output. The standard deviation in latency output was set to 10% of the Wi-Fi traffic load in all experiments. In the case of continuous and legacy CSMA/CA, PRR decreases as the Wi-Fi traffic load increases. Since Wi-Fi uses wideband, Wi-Fi devices do not detect Zigbee signals using narrowband and low-power transmission. This prevents channel coordination between Wi-Fi and Zigbee, which causes interference. In continuous mode, most of the lost packets are corrupted by IEEE 802.11 interference. Zigbee with CSMA/CA can detect the Wi-Fi signal and perform backoff. This delays the transmission of Zigbee packets, and some packets are dropped in the buffer if the backoff time becomes long. In addition, it still experiences significant packet loss caused by IEEE 802.11 interference. ECC and CTC-CSMA/CA perform Zigbee transmission only in guaranteed white space, thus they are not affected by interference from Wi-Fi. They show PRRs of over 97.48%.

**Figure 6:**
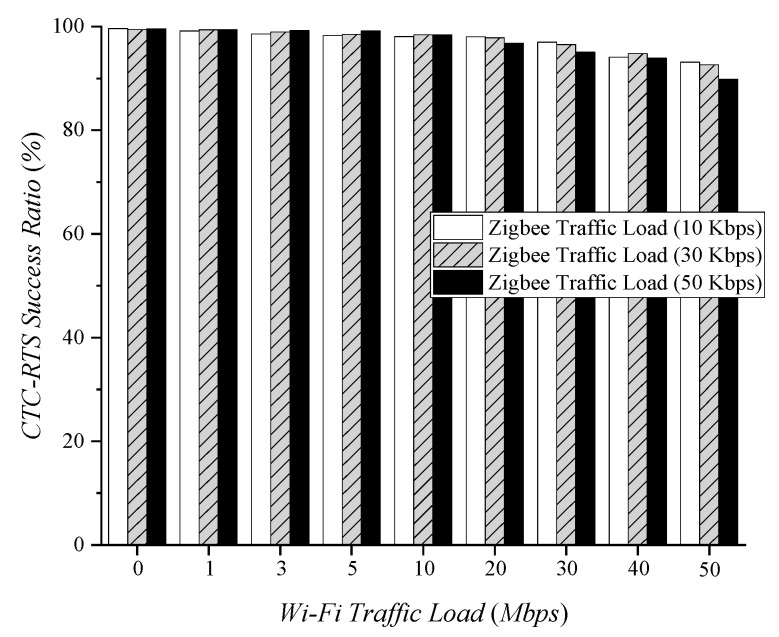
CTC-RTS success ratio according to Wi−Fi traffic load.

Figure 6 shows the success ratio of CTC-RTS frames based on different Wi-Fi traffic loads. Zigbee performs carrier sensing before transmitting a CTC-RTS frame, but it is still affected by Wi-Fi interference since sharing channels are not coordinated. Therefore, as Wi-Fi traffic increases, the success ratio of the CTC-RTS frames decreases. Since the CTC-RTS frame is short and transmitted once per beacon interval, it has less loss than legacy CSMA/CA test results. High Zigbee traffic reduces the channel capacity that Wi-Fi devices use. This results in increasing the probability of CTC-RTS frame loss. In the experimental results, the CTC-RTS frames showed a success ratio of 89.82% or more. This means that Zigbee requests for channel coordination can be reliably delivered to Wi-Fi.

### 4.2. Interference-Free Period Allocation

**Figure 7:**
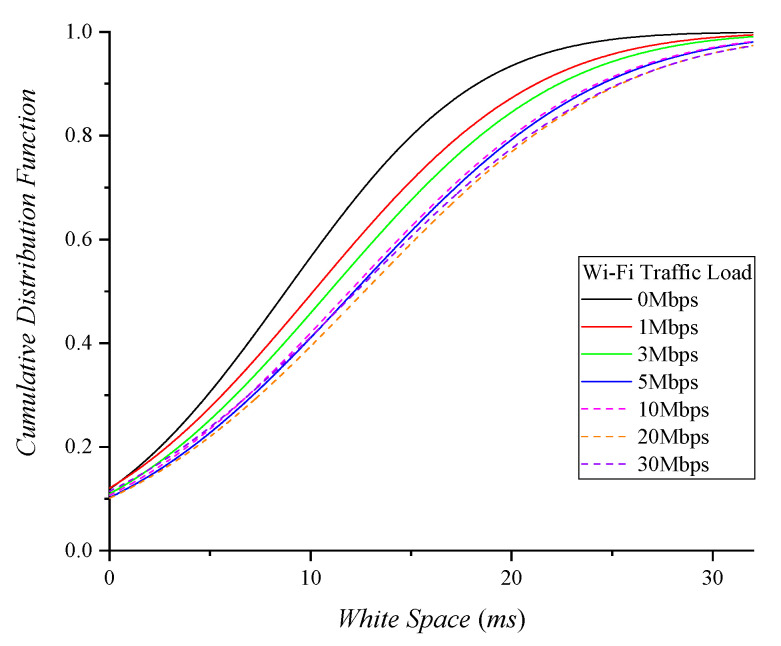
ECC white space with various Wi−Fi traffic loads.

ECC and CTC-CSMA/CA secure an interference-free period via white space allocation. Assuming that a fixed volume of Wi-Fi traffic occurs randomly in a fixed period, we first examine the white space distribution of ECC. Figure 7 shows the cumulative distribution of white space determined by ECC with different Wi-Fi traffic loads. When the Wi-Fi traffic range is 0 to 5 Mbps, the larger the Wi-Fi traffic, the longer white space is provided. However, from 5 to 30 Mbps, there is no correlation between the volume of Wi-Fi traffic and the length of white space. The negative feedback loop in ECC [4] determines the next white space based on the previous traffic load. The white space in ECC is more affected by the variability of the traffic than by the capacity of the traffic. Hence, ECC cannot accurately reflect the changes in Wi-Fi traffic load and the required white space lengths for Zigbee.

**Figure 8:**
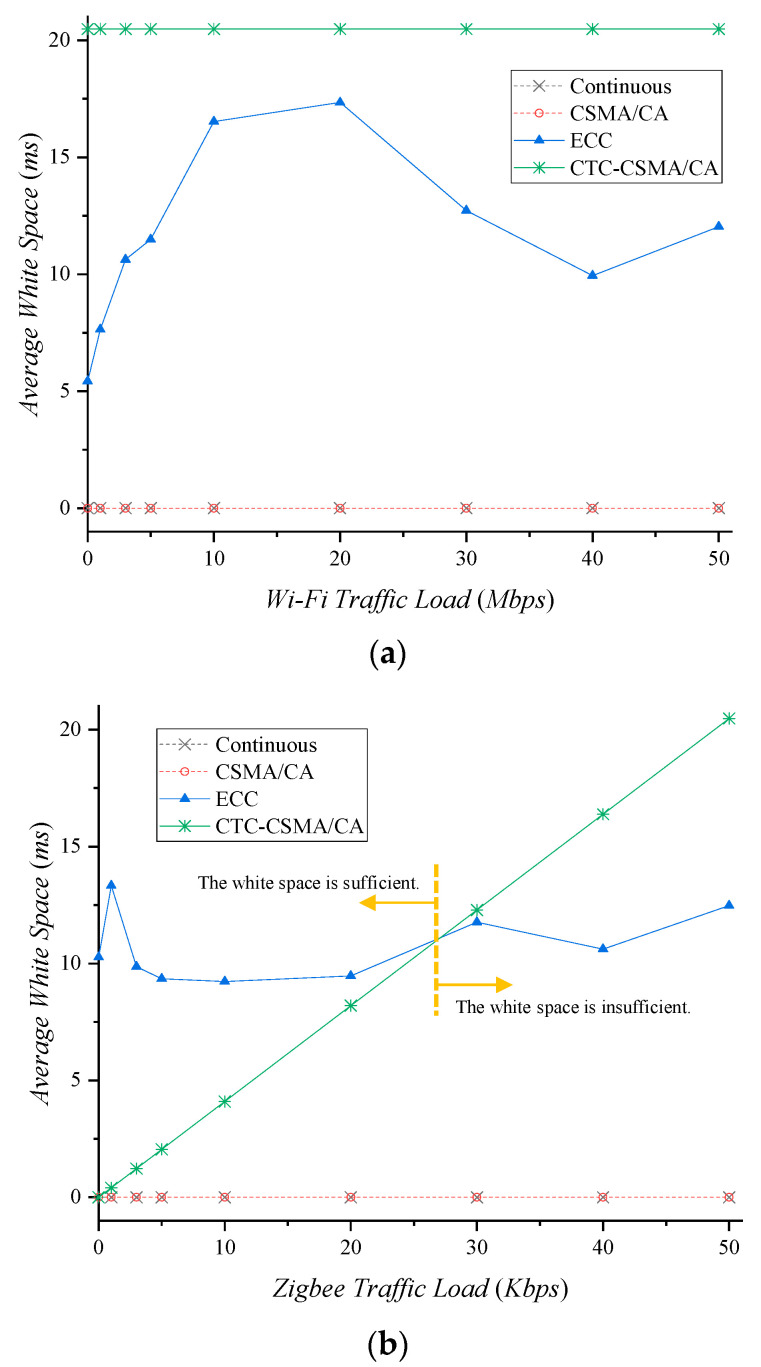
Average white space according to traffic load. (**a**) Wi−Fi traffic load, (**b**) Zigbee traffic load.

Depending on the amount of traffic in the network, we compared the interference-free periods that different schemes provide. Figure 8a shows the average white space according to Wi-Fi traffic load when Zigbee transmits packets at 50 Kbps. Continuous and legacy CSMA/CA do not have any white space because they do not coordinate the shared channel with Wi-Fi. ECC has an average white space of 11.54 ms in the experiment. To deal with 50 Kbps Zigbee traffic, 20.48 ms of white space is necessary. This means ECC can protect 56.28% of Zigbee traffic via the white space. In the case of CTC-CSMA/CA, Zigbee requests the white space it needs, and the Wi-Fi AP determines whether to allow it by considering the Wi-Fi traffic status. In this experiment, since Zigbee transmits packets at 50 Kbps, it requested a 20.48 ms white space in every beacon period. The CTC-RTS frame may be lost due to Wi-Fi interference, as in Figure 6, but Zigbee retransmits the CTC-RTS frame, including the previous white space request, in the next beacon period. Therefore, the white space that is not allocated due to the CTC-RTS frame loss or a denial of white space allocation by the Wi-Fi AP is delayed but is eventually secured. In CTC-CSMA/CA, Zigbee can secure the necessary white space regardless of Wi-Fi traffic load.

Figure 8b shows the average white space according to Zigbee traffic when Wi-Fi generates 40 Mbps of traffic on average. Since Wi-Fi traffic has a fixed standard deviation in latency output, ECC provides an average white space of around 10 ms per experiment. This is sufficient if Zigbee traffic is low, but in many cases, packet loss may occur due to insufficient white space. In CTC-CSMA/CA, the white space increases linearly as Zigbee traffic increases, because Zigbee requests more white space. Therefore, CTC-CSMA/CA can effectively use the channel without insufficiency or waste.

### 4.3. Throughput Gain and Loss

**Figure 9:**
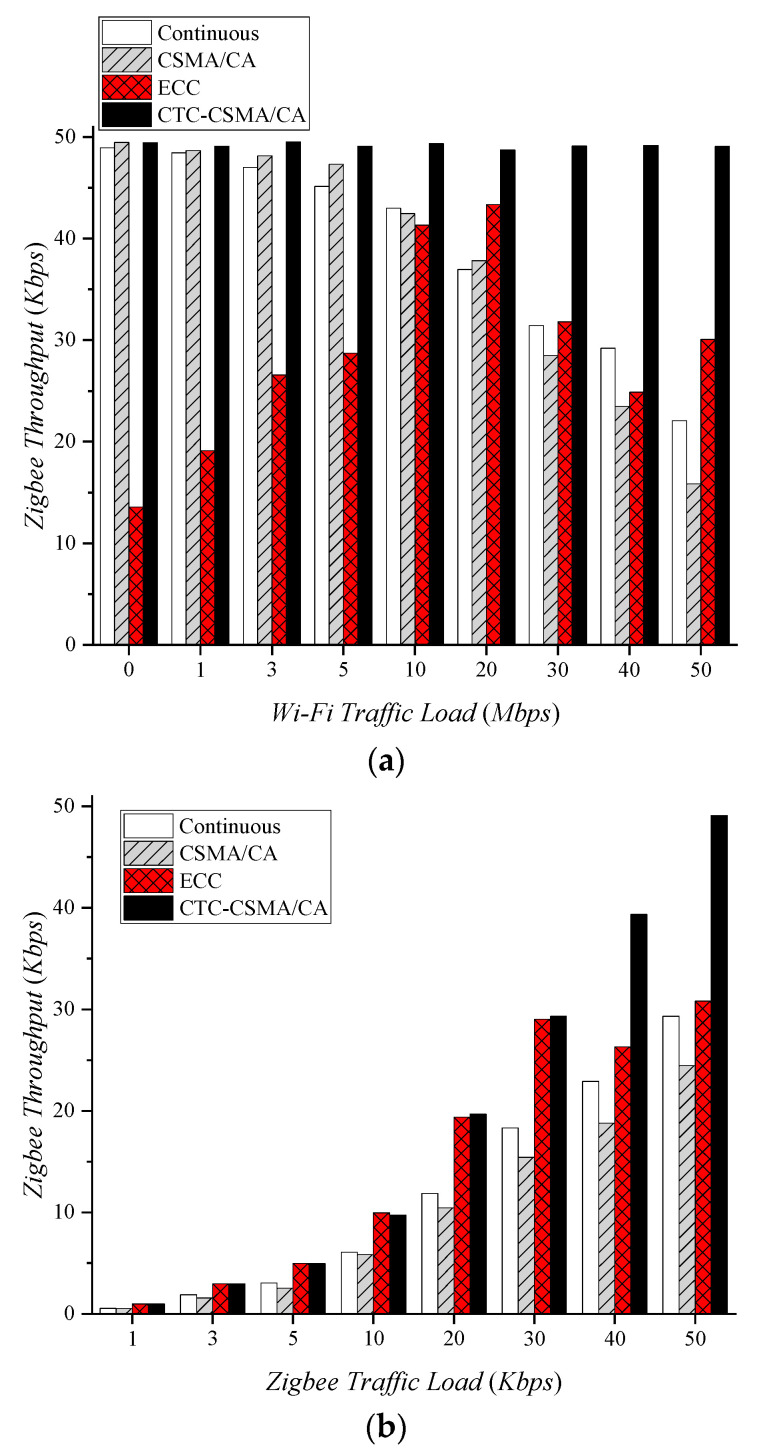
Zigbee throughput according to traffic load. (**a**) Wi−Fi traffic load, (**b**) Zigbee traffic load.

In this section, we investigate throughput gain and loss via white space allocation. To examine the effect of Wi-Fi traffic load on Zigbee throughput, Zigbee generates traffic at 50 Kbps. Since continuous and legacy CSMA/CA have no white space, high Wi-Fi traffic causes more interference to Zigbee. As shown in Figure 5, the interference decreases the PRR of Zigbee. Figure 9a shows that the Zigbee throughput decreases as the Wi-Fi traffic load increases in continuous and legacy CSMA/CA.

We assume ECC and CTC-CSMA/CA transmit Zigbee packets only during white space. ECC shows the highest white space of 17.34 ms with a 20 Mbps traffic load (Figure 9a), since the white space is determined by the randomness of Wi-Fi traffic. Zigbee transmits packets without interference during this white space, resulting in a throughput of 43.45 Kbps. It is still not enough white space to deal with 50 Kbps traffic. In CTC-CSMA/CA, Zigbee provides nearly a 50 Kbps throughput because it almost satisfies the white space requirement. As in Figure 9a, CTC-CSMA/CA obtains 25.66%, 29.52%, and 70.62% throughput gains compared to continuous, CSMA/CA, and ECC, respectively.

To investigate the effect of Zigbee traffic load on Zigbee throughput, Wi-Fi traffic was fixed at 40 Mbps. Figure 9b shows Zigbee throughput according to Zigbee traffic load. In the case of continuous and CSMA/CA, throughput increases as Zigbee traffic increases. However, since packets are lost due to Wi-Fi interference, not all of the traffic can be successfully delivered. Continuous and CSMA/CA lost 40.53% and 48.43% of average Zigbee throughput, respectively. Since ECC determines the white space regardless of the amount of Zigbee traffic, it provides white space of about 10 ms, as in Figure 8b. When Zigbee traffic load is low, it processes all the traffic, but when Zigbee traffic load is high, it cannot deal with all the traffic. If the Zigbee traffic load is more than 30 Kbps, Zigbee suffers a 25.25% throughput loss due to dropped packets from the buffer. CTC-CSMA/CA can deal with all traffic generated by Zigbee. This represents a very low throughput loss of 1.87%, yielding 66.18%, 96.08%, and 25.48% throughput gains over continuous, legacy CSMA/CA, and ECC, respectively.

**Figure 10:**
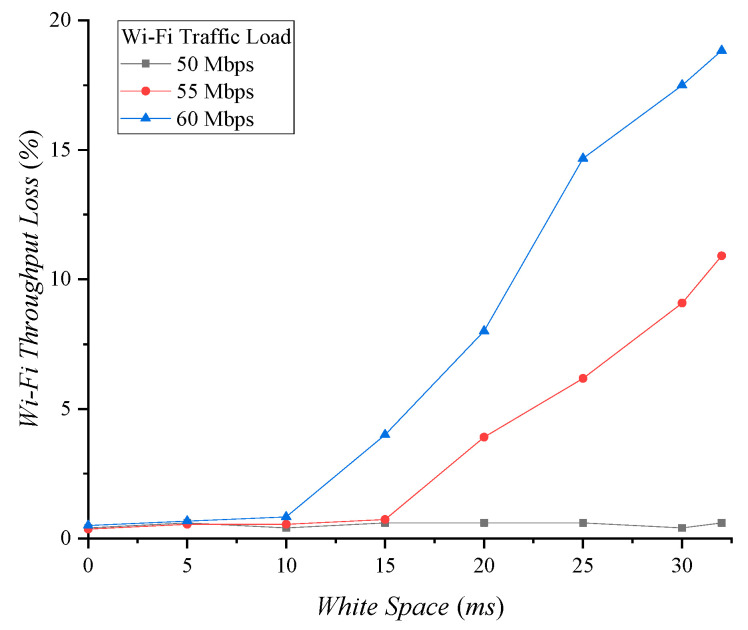
Wi-Fi throughput loss according to white space.

Figure 10 shows Wi-Fi throughput loss due to white space allocation. When the Wi-Fi traffic load is less than 50 Mbps, Wi-Fi throughput loss due to white space allocation hardly occurs. With a 55 Mbps Wi-Fi traffic load, Wi-Fi throughput is lost when the white space exceeds 15 ms. To secure white space, the available Wi-Fi bandwidth is reduced, which incurs throughput loss. As Wi-Fi traffic load increases, the throughput loss due to white space increases. We need to consider whether to allocate white space by considering the priorities of Wi-Fi and Zigbee.

## 5. Conclusions

In this paper, we achieved CSMA/CA between Wi-Fi and Zigbee for explicit channel coordination. The proposed scheme, CTC-CSMA/CA, enables the exchange of RTS/CTS frames between incompatible physical layers via bidirectional CTC. By means of CTC-CSMA/CA, a Wi-Fi AP can allocate white space more accurately than by ECC assigning the white space without Zigbee feedback (e.g., RTS). Empirical evaluation validates high throughput improvement for Zigbee through accurate white space allocation. In particular, CTC-CSMA/CA shows higher efficiency when Wi-Fi and Zigbee traffic dynamics are large. It is expected to be a major application case using CTC. In the future, further research is needed on explicit channel coordination considering traffic priorities when Wi-Fi and Zigbee traffic is dense.
